# Intraoperative demonstration of a check-valve mechanism in a spinal extradural arachnoid cyst with nerve root incarceration

**DOI:** 10.1093/jscr/rjag1020

**Published:** 2026-11-04

**Authors:** Takashi Dezawa, Katsunori Fukutake, Keiji Hasegawa, Daisuke Kamakura, Naobumi Tochigi, Hiroshi Takahashi

**Affiliations:** Department of Orthopedic Surgery, Toho University Omori Medical Center, 6-11-1 Omorinishi, Ota-ku, 143-8541, Tokyo, Japan; Department of Orthopedic Surgery, Toho University Omori Medical Center, 6-11-1 Omorinishi, Ota-ku, 143-8541, Tokyo, Japan; Department of Orthopedic Surgery, Toho University Omori Medical Center, 6-11-1 Omorinishi, Ota-ku, 143-8541, Tokyo, Japan; Department of Orthopedic Surgery, Toho University Omori Medical Center, 6-11-1 Omorinishi, Ota-ku, 143-8541, Tokyo, Japan; Department of Surgical Pathology, Toho University Omori Medical Center, 6-11-1 Omorinishi, Ota-ku, 143-8541, Tokyo, Japan; Department of Orthopedic Surgery, Toho University Omori Medical Center, 6-11-1 Omorinishi, Ota-ku, 143-8541, Tokyo, Japan

**Keywords:** arachnoid cyst, spinal extradural arachnoid cyst, check-valve mechanism, dural defect, nerve root incarceration

## Abstract

Spinal extradural arachnoid cysts (SEDACs) are rare lesions thought to arise from a dural defect associated with a check-valve mechanism, although direct intraoperative evidence is limited. We report a 17-year-old female presenting with persistent low back pain and bilateral lower extremity numbness. Magnetic resonance imaging demonstrated a cystic lesion at the Th12–L1 level displacing the dural sac, while computed tomography myelography failed to identify a communicating tract. Surgical exploration was performed due to persistent symptoms and a suspected dynamic mechanism. Intraoperatively, a dural defect at the nerve root sleeve with nerve root incarceration was identified. Cerebrospinal fluid leakage ceased after closure of the defect, confirming a check-valve mechanism. The cyst was resected and the defect repaired. The patient’s symptoms resolved completely, with no recurrence at 2-year follow-up. This case provides direct intraoperative evidence of the proposed pathophysiology of SEDAC.

## Introduction

Spinal epidural arachnoid cysts are rare lesions classified as Type I in the Nabors classification and may cause back pain, radiculopathy, or compressive myelopathy [1]. They are thought to arise from dural defects with a check-valve mechanism that allows cerebrospinal fluid to accumulate within the cyst. This case provides direct intraoperative evidence of a check-valve mechanism caused by nerve root incarceration at a dural defect.

## Case report

The patient was a 17-year-old female who developed low back pain and bilateral lower extremity numbness without a trigger 3 months ago. She visited a nearby physician for a detailed examination, and was suspected of having a spinal cord tumor and referred to our hospital. She had low back pain and numbness involving both lower extremities, which was increased by a forward leaning posture. No motor weakness, bladder dysfunction, or bowel dysfunction was present. Magnetic resonance imaging (MRI) of lumbar vertebrae showed T1 low and T2 high well-circumscribed cystic lesions located posterior to the dural sac at Th12 and L1. The lesions compressed and displaced the dural sac (Fig. 1A–C). Computed tomography (CT) after myelography showed scalloping from the lamina to the Th12 vertebral arch. No cystic lesion or communicating foramen was found (Fig. 2A and B). Th12 and L1 vertebral arches were resected to the inside of the facet joint in surgery. Cystic lesions were located in the dorsal part of dural sac as a mass and excluded the dura to the ventral parts (Fig. 3A). Blunt detachment of cysts from the spinal cord was performed from the cranial end (Fig. 3B). Dural incision was used under the assumption of a dural defect on the left side. After dural incision with preservation of the arachnoid mater, the dura was observed from the dorsal side. An ~7-mm fissure and nerve root incarceration were found in the caudal region of the Th12 pedicle branch (Fig. 4A). We concluded that incarceration of nerve root at the dural defect created a check-valve mechanism, leading to progressive cyst enlargement. The nerve root was reduced, the dura was sutured, and the communicating foramen was closed, after which the cysts were resected as much as possible (Fig. 4B). No cerebrospinal fluid leak occurred after dural suture. Histopathological examination revealed that the inner layer consisted of meningothelial cells forming the cyst lining. The outer layer consisted of fibrous connective tissue, consistent with an arachnoid cyst (Fig. 5). The patient recovered without complication and was discharged to home 2 weeks after surgery. Preoperative low back pain and bilateral lower extremity numbness gradually improved, and at 5 months postoperatively, MRI showed no evidence of cyst recurrence (Fig. 6). At 2-year follow-up, the patient’s symptoms had resolved.

**Figure 1:**
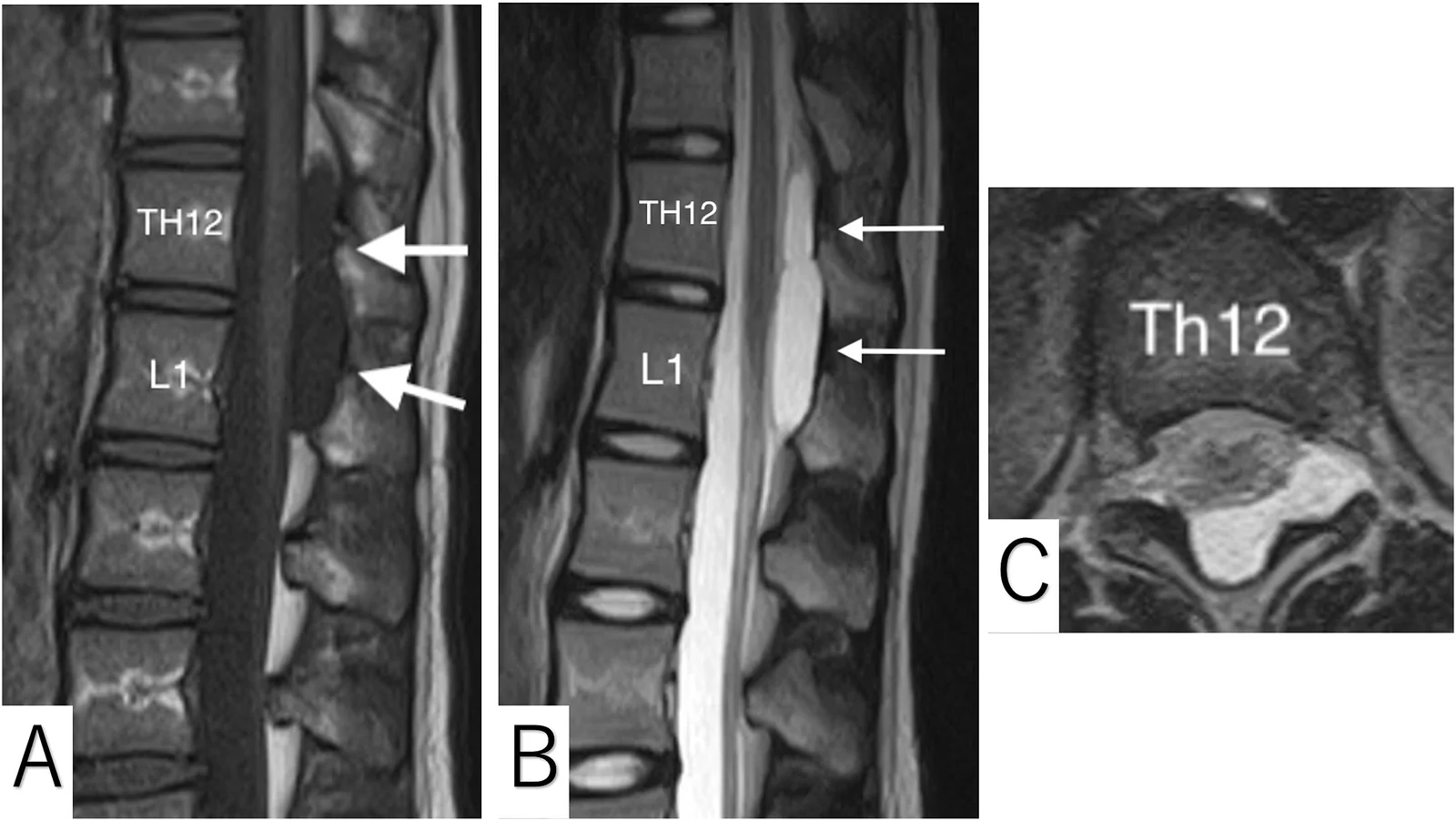
MRI of lumbar vertebrae showed T1 low (A) and T2 high (B and C) well-circumscribed cystic lesions dural to the dural sac at Th12 and L1. The lesions displaced the dural sac ventrally and toward the right side.

**Figure 2:**
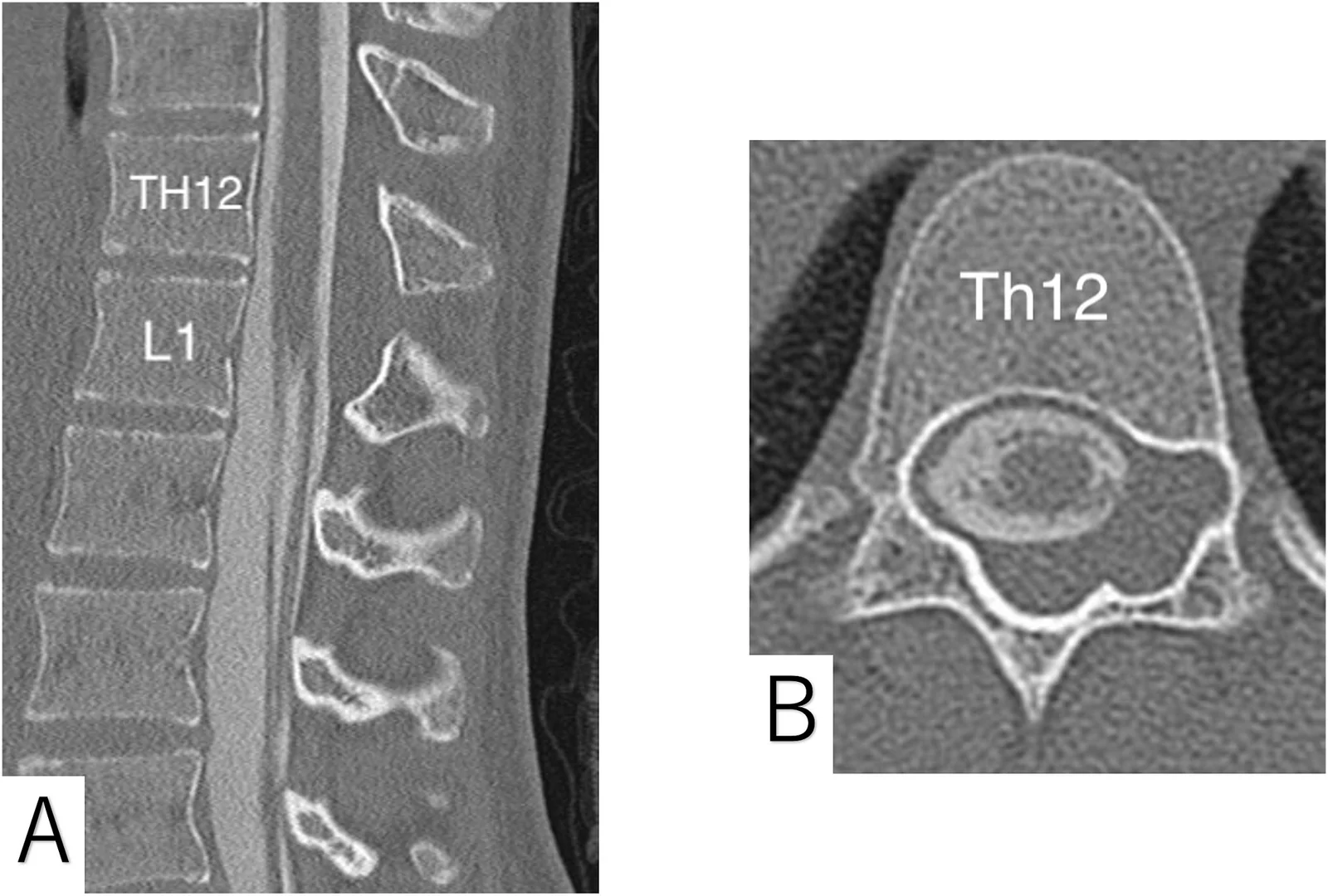
CT after myelography showed scalloping from the lamina to the Th12 vertebral arch. No cystic lesion or communicating foramen was found (A and B).

**Figure 3:**
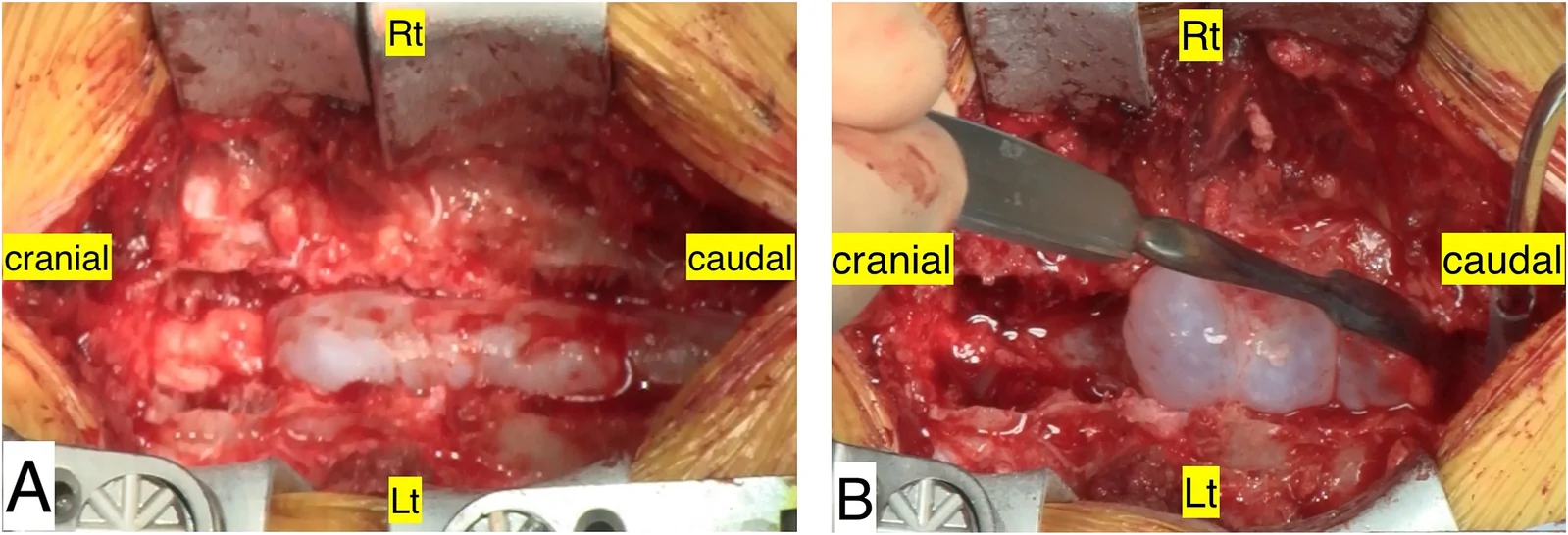
Intraoperative findings after laminectomy. Cystic lesions were located dorsal to the dural sac as a mass and displaced the dural sac ventrally (A and B).

**Figure 4:**
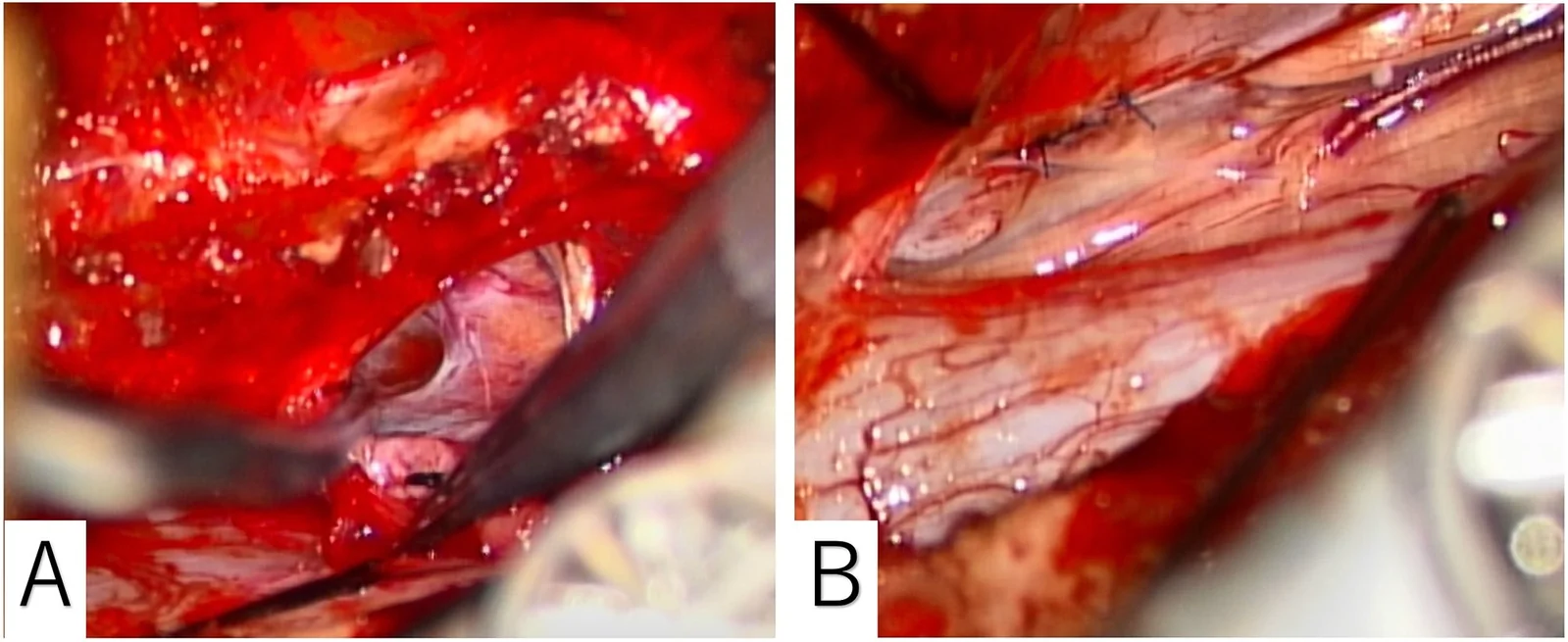
Intraoperative during reduction of the incarcerated nerve root and repair of the dural defect. An ~7-mm fissure and nerve root incarceration were found in the caudal region of the Th12 pedicle branch (A). The nerve root was reduced, the dura was sutured, and the communicating foramen was closed, after which the cysts were resected as much as possible (B).

**Figure 5:**
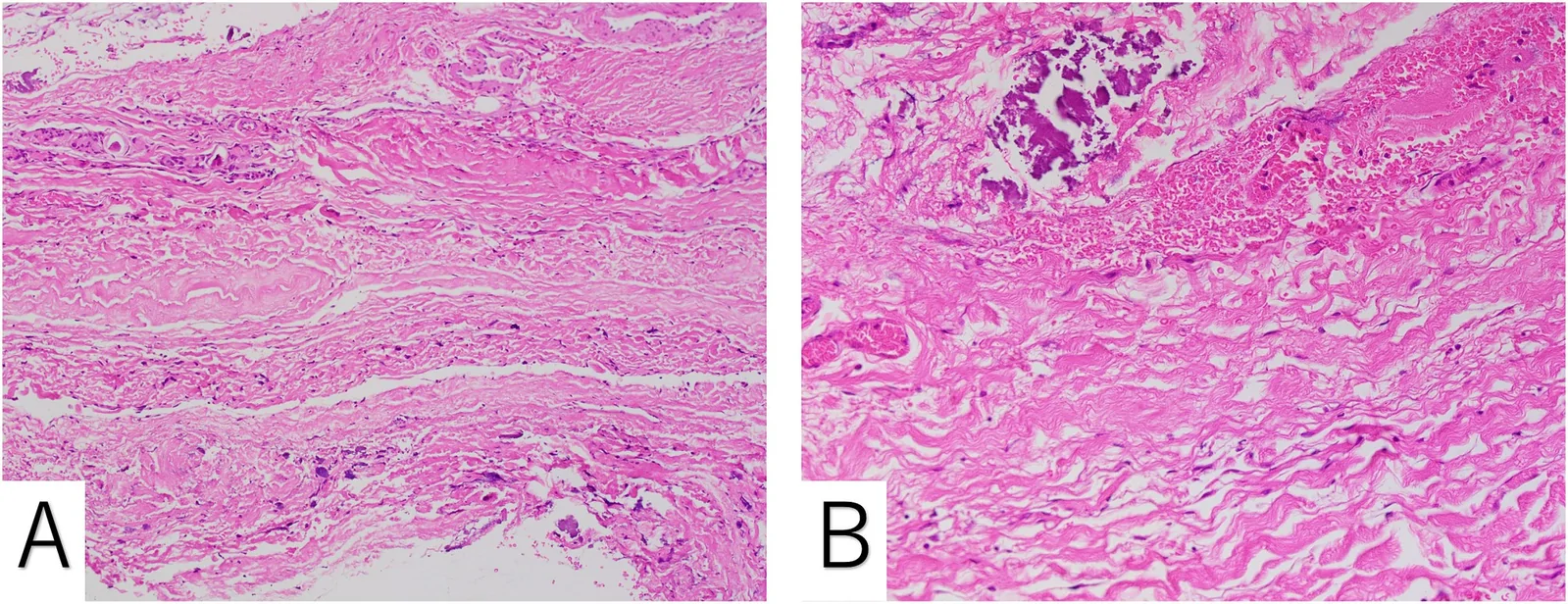
Histopathological examination. The inner layer consisted of meningothelial cells forming the cyst lining (A). The outer layer consisted of fibrous connective tissue, consistent with an arachnoid cyst (B).

**Figure 6:**
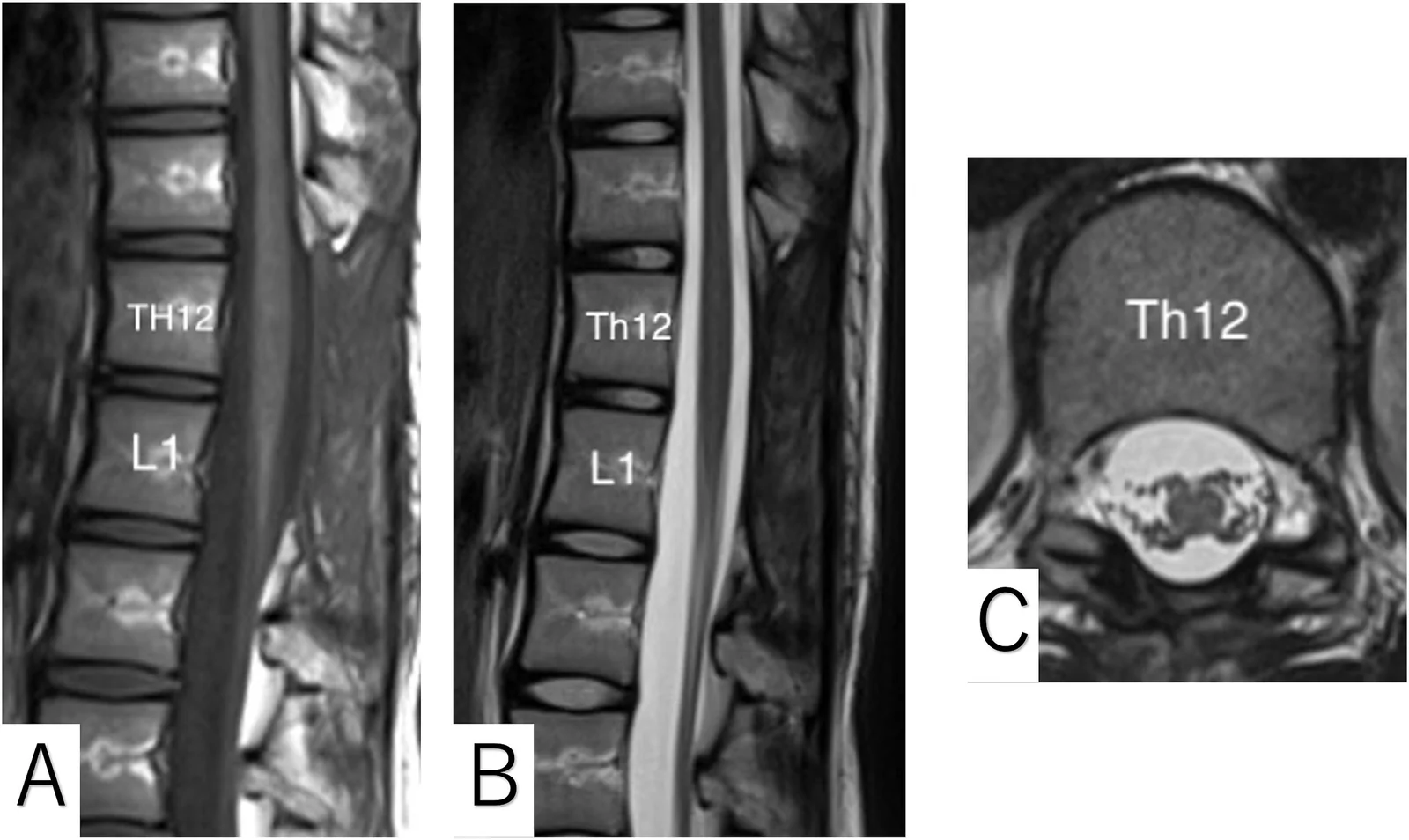
MRI at 5 months postoperatively. Sagittal T1-weighted MRI (A), Sagittal T2-weighted MRI (B), Axial T2-weighted MRI (C). Follow-up imaging showed no signs of cyst recurrence.

## Discussion

Spinal extradural arachnoid cysts (SEDACs) are relatively rare lesions classified as Nabors type I and may present with a spectrum of symptoms, including low back pain, radiculopathy, and compressive myelopathy [1, 2]. The major causes of the disease are idiopathic, but also include trauma, infection, meningitis, subarachnoid hemorrhage, and postoperative arachnoiditis [3–5]. Surgical treatment is generally recommended for symptomatic patients, particularly those presenting with neurological deficits or progressive symptoms. In the present case, the patient presented with persistent bilateral lower extremity numbness without motor deficits. However, several factors supported surgical intervention. Imaging studies demonstrated a well-defined cystic lesion at the Th12–L1 level causing displacement of the dural sac, suggesting a potential risk of progressive compression. In addition, symptom exacerbation with forward flexion suggested a dynamic mechanism such as a check-valve phenomenon. Conservative management was considered unlikely to resolve the underlying pathology, as SEDACs associated with a dural defect may progressively enlarge due to continuous cerebrospinal fluid (CSF) inflow. Therefore, surgical treatment was considered appropriate to alleviate sensory symptoms and prevent further neurological deterioration. Even though there was no history of major trauma or congenital defect, multiple microtraumas can cause a dural tear near the nerve root sleeves because of tension across the movable dural sac and relatively fixed nerve roots [6–8]. In the present case, intraoperative findings revealed a dural defect at the nerve root sleeve with incarceration of the nerve root. This supports the hypothesis that mechanical entrapment contributes to the formation of a check-valve mechanism, in which a one-way valve forms between the subarachnoid space and cysts, allowing CSF inflow and cyst expansion [9]. Direct visualization of both the dural defect and nerve root incarceration provides important insight into the pathophysiology of SEDAC. Sangala *et al.* reported nerve root prolapse into a spinal arachnoid cyst causing radiculopathy [10]. In contrast, the present case directly demonstrated incarceration of a nerve root at the dural defect itself, providing intraoperative evidence supporting the proposed check-valve mechanism. Preoperative identification of the communicating tract or dural defect remains challenging. In this case, neither MRI nor CT myelography could detect the defect. Although subtle displacement of the dural sac suggested the lesion side, precise localization was not possible. Advanced imaging modalities such as dynamic or cine MRI may improve detection of CSF flow and should be considered in future cases [11]. Regarding surgical strategy, the procedures include subarachnoid release, communicating foramen closure, partial resection, and total resection [12]. Although motion-preserving techniques are generally preferred, total laminectomy was performed in this case to ensure sufficient exposure for identification and repair of the dural defect. The extent of resection was limited to preserve spinal stability. There are reports of relapse and adhesion after subarachnoid release, and if possible, total resection is beneficial from the viewpoint of relapse prevention [13–15]. In this patient, total resection was feasible due to mild adhesion, and no recurrence was observed following dural repair. In conclusion, this case highlights the importance of identifying and repairing a dural defect with nerve root incarceration as a key mechanism in SEDAC formation, and suggests that surgical intervention may be beneficial in patients with persistent sensory symptoms attributable to SEDAC.
